# On Forgotten Topological Indices of Some Dendrimers Structure

**DOI:** 10.3390/molecules22060867

**Published:** 2017-05-24

**Authors:** Yasir Bashir, Adnan Aslam, Muhammad Kamran, Muhammad Imran Qureshi, Adnan Jahangir, Muhammad Rafiq, Nargis Bibi, Nazeer Muhammad

**Affiliations:** 1 Department of Mathematics, COMSATS Institute of Information Technology, Wah Cantt 47040, Pakistan; yasirbashir@ciitwah.edu.pk (Y.B.); m.kamran@ciitwah.edu.pk (M.K.); adnan.jahangir@ciitwah.edu.pk (A.J.); rafiq@ciitwah.edu.pk (M.R.); 2 Department of Natural Sciences and Humainities, University of Engineering and Technology, Lahore (RCET) 54890, Pakistan; adnanaslam15@yahoo.com; 3 Department of Mathematics, COMSATS Institute of Information Technology, Vehari 61100, Pakistan; imranqureshi18@gmail.com; 4 Department of Computer Science, Fatima Jinnah Women University, Rawalpindi 46000, Pakistan; nargis@fjwu.edu.pk

**Keywords:** forgotten index, porphyrin dendrimers, poly(propyl) ether imine dendrimer

## Abstract

A series of previously conducted experiments pertaining to various chemicals and drugs uncover a natural linkage between the molecular structures and the bio-medical and pharmacological characteristics. The forgotten topological index computed for the molecular structures of various chemical compounds and drugs has proven significant in medical and pharmaceutical fields by predicting biological features of new chemical compounds and drugs. A topological index can be considered as the transformation of chemical structure into a real number. Dendrimers are highly-branched star-shaped macromolecules with nanometer-scale dimensions. Dendrimers are defined by three components: a central core, an interior dendritic structure (the branches), and an exterior surface with functional surface groups. In this paper, we determine forgotten topological indices of poly(propyl) ether imine, porphyrin, and zinc–porphyrin dendrimers.

## 1. Introduction

We are living in an era where every day sees better innovation than the previous, with the same trend in the enhancement and innovation in the production of different types of medicines, chemical compounds, and drugs for the improved health of humans and other living species on the planet. It requires a great amount of time and money to test these drugs and chemical compounds to determine their pharmacological, chemical, and biological characteristics using expensive equipment, which in turn makes the task more cumbersome. This task of evaluating the biological behavior and existence of side effects of chemical compounds becomes more difficult in countries with economic imbalance. In this regard, computing different types of topological indices has provided the indicators of such medicinal behaviour of several compounds and drugs. The computation method of topological indices has proven its worth by yielding medical information of drugs with less use of chemical-related equipment.

Molecules and molecular compounds are often modeled by molecular graphs. A molecular graph is a representation of the structural formula of a chemical compound in terms of graph theory, whose vertices correspond to the atoms of the compound and edges correspond to chemical bonds. A graph G(V,E) with vertex set *V* and edge set *E* is connected if there exists a connection between any pair of vertices in *G*. For a graph *G*, the degree of a vertex *v* is the number of edges incident with *v* and denoted by $d_{v}$.

A graph can be recognized by a numeric number, a polynomial, a drawing, a sequence of numbers, or a matrix. A topological index is a numerical quantity associated with a graph that characterizes the topology of the graph and is invariant under graph automorphism. Among various topological indices, degree-based topological indices are the most important and widely used. These have great application in chemical graph theory. Since the 1970s, two degree-based graph invariants have been extensively studied. These are the first Zagreb index $M_{1}$ and the second Zagreb index $M_{2}$, defined as

$$M_{1}\left(G\right)=\sum_{v\in V(G)}{\left(d_{v}\right)}^{2}.$$

$$M_{2}\left(G\right)=\sum_{uv\in E(G)}\left(d_{u}d_{v}\right).$$

Details on the two Zagreb topological indices can be found in [1]. The Zagreb index $M_{1}$ was first encountered in a paper published in 1972 [1], where a series of approximate formulas for total π-electron energy *E* were deduced. By means of these formulas, several structural details have been identified, upon which *E* depends. Among these was the sum of squares of the vertex degrees of the underlying molecular graph are discussed. In the approximate formulas for *E*, there was also a term equal to the sum of cubes of the vertex degrees [1]. However, the latter term was completely ignored by scholars doing research on degree-based topological indices with various transformations [3,4,5,6,7,8,9,10,11,12,13]. Recently, Furtula and Gutman [14] have restudied this term to establish some basic properties, and have also demonstrated that the predictive ability of this term is similar to that of the first Zagreb index with respect to entropy and acetic factors of the molecules are discussed. Both first Zagreb index and this index yield are used to observe the correlation coefficients with larger value than 0.95. They named this term the “forgotten topological index” or “*F*-index”, and it is defined as

(1) $$F\left(G\right)=\sum_{v\in V(G)}{\left(d_{v}\right)}^{3}=\sum_{uv\in E(G)}\left[{\left(d_{u}\right)}^{2}+{\left(d_{v}\right)}^{2}\right].$$

For more detail on the “*F*-index”, we refer to the articles [15,16,17].

Analogous to other topological polynomials, the *F*-polynomial of graph *G* is also defined as:

(2) $$F\left(G,x\right)=\sum_{uv\in E(G)}x^{\left[{\left(d_{u}\right)}^{2}+{\left(d_{v}\right)}^{2}\right]}$$

Dendrimers are constructed by hyperbranched macromolecules with a fully-tailored architecture. They can be arranged in a composed manner by either convergent or divergent form. Dendrimers have a huge range of applications in all branches of chemistry, especially in host–guest reactions and self-assembly procedures. Dendrimers are used in the formation of nanotubes, nanolatex, chemical sensors, micro/macro capsules, coloured glass, modified electrodes, and photon funnels such as artificial antennas [18]. Because dendrimers are widely used in different applied fields, the study of nanostar dendrimers has received a great deal of attention in both chemical and mathematical literature. For other different applications regarding dendrimers, we refer to [18]. Until now, the study of the *F*-index for special chemical and nano-structures has been largely limited. Thus, we have been attracted to studying the mathematical properties of the *F*-index and its polynomial version of some dendrimers. In this paper, we determine *F*-index and polynomial of poly(propyl) ether imine, porphyrin, and zinc–porphyrin dendrimers.

In order to find the number of edges of the arbitrary graph, the following lemma is used.

**Lemma** **1.** *Let G be a graph. Then* $\sum_{v\in V(G)}d_{v}=2|E|$.

## 2. F-Index and Polynomial of Poly(Propyl) Ether Imine Dendrimer

**Figure 1:**
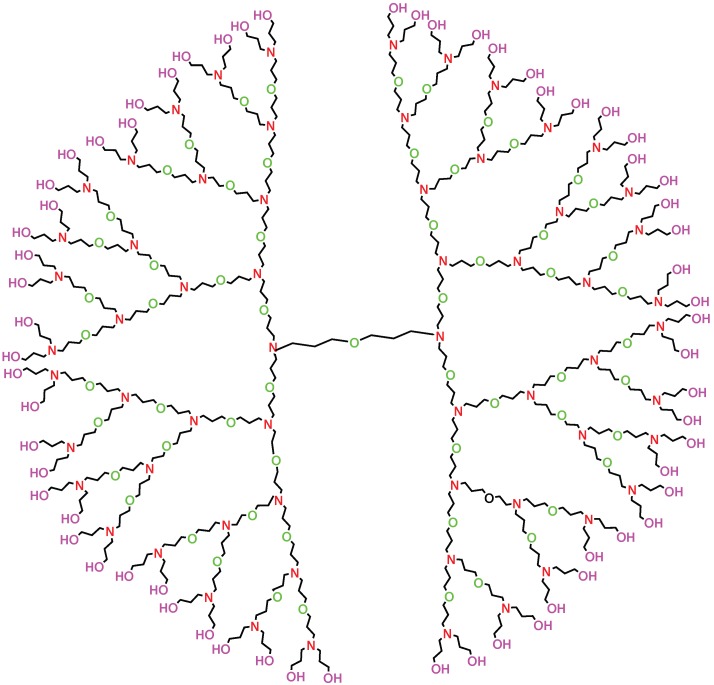
Molecular structure of poly(propyl) ether imine (PETIM) dendrimer with n=5.

Polynomial of Poly(Propyl) Ether Imine (PETIM) dendrimer starts growing three-dimensionally from the oxygen as the core and branches out at each tertiary nitrogen, which is separated by eight-bond spacer for each generation of the dendrimer. Consider the molecular graph *G* of PETIM dendrimer of generation $G_{n}$ with *n* growth stages, where n≥1 (see Figure 1). The graph of PETIM dendrimer consists of four branches and a central core consisting of eight edges. In each branch, we have $8+2\times 8+2^{2}\times 8+\cdots +2^{n-2}\times 8+4\times 2^{n-1}=6\times 2^{n}-8$ edges. A simple calculation shows that the total number of edges in *G* are $24\times 2^{n}-24$. Since *G* is a tree, it follows directly that the number of vertices of *G* are $24\times 2^{n}-23$.

In *G*, the total number of vertices of degree 1 are the leaves, which are $4\times 2^{n-1}=2^{n+1}$ in number. The vertices of degree 3 are $4\left(1+1\times 2+1\times 2^{2}+\cdots +1\times 2^{n-2}\right)+2=2^{n+1}-2$. The remaining $24\times 2^{n}-23-\left(2^{n+1}-2\right)-2^{n+1}=20\times 2^{n}-21$ vertices are of degree 2. Let $e_{ij}$ denote the number of edges of *G* connecting vertices of degrees *i* and *j*. Clearly, $e_{ij}=e_{ji}$. Let us denote the number of edges connecting vertices of degrees *i* and *j* in each branch of the dendrimer by $e_{ij}^{\prime }$. It is easy to see that the central core consists of six edges $e_{ij}$ with i=j=2 and two edges $e_{ij}$ with i=2 and j=3. Then, we have $e_{12}=4e_{12}^{\prime }$, $e_{22}=4e_{22}^{\prime }+6$ and $e_{23}=e_{23}^{\prime }+2$. For n>1, we can calculate $e_{12}^{\prime }=2^{n-1}$, $e_{22}^{\prime }=\left(6+2\times 6+\cdots +2^{n-2}\times 6\right)+2\times 2^{n-1}=4\times 2^{n}-6$, $e_{23}^{\prime }=6\times 2^{n}-8-2^{n-1}-\left(4\times 2^{n}-6\right)=3\times 2^{n-1}-2$. Therefore, we have $e_{12}=2^{n+1}$, $e_{22}=16\times 2^{n}-18$, and $e_{23}=6\times 2^{n}-6$. Now, we compute forgotten index and polynomial for the poly(propyl) ether imine (PETIM) dendrimer in the following theorem.

**Theorem** **1.** *Let G be the molecular graph of PETIM dendrimer. Then*

$$F\left(G\right)=216\times 2^{n}-222.$$

$$F\left(G,x\right)=2^{n+1}x^{5}+\left(16\times 2^{n}-18\right)x^{8}+\left(6\times 2^{n}-6\right)x^{13}.$$

**Proof.**  Let *G* be a graph of PETIM dendrimer. The vertex set V(G) is divided into three sets based on the degree of the vertices. The first vertex set $V_{1}\left(G\right)$ consists of $2^{n+1}$ vertices of degree 1. The second vertex set $V_{2}\left(G\right)$ consists of $20\times 2^{n}-21$ vertices of degree 2. The third vertex set $V_{3}\left(G\right)$ consists of $2^{n+1}-2$ vertices of degree 3. From (1), the F-index of *G* is given by

$$\begin{array}{cc} F(G) & =\sum_{v\in V_{1}\left(G\right)}{\left(d_{v}\right)}^{3}+\sum_{v\in V_{2}\left(G\right)}{\left(d_{v}\right)}^{3}+\sum_{v\in V_{3}\left(G\right)}{\left(d_{v}\right)}^{3}\\ & =216\times 2^{n}-222 \end{array}$$

Similarly, the edge partitions of *G*, based on the degree of end vertices, are defined as $E_{1}\left(G\right)=\left\{e=uv\in E\left(G\right):d_{u}=1\;\mathrm{and}\;d_{v}=2\right\}$, $E_{2}\left(G\right)=\left\{e=uv\in E\left(G\right):d_{u}=d_{v}=2\right\}$ and $E_{3}\left(G\right)=\left\{e=uv\in E\left(G\right):d_{u}=2\;\mathrm{and}\;d_{v}=3\right\}$. Therefore, we have $|E_{1}\left(G\right)|=2^{n+1}$, $|E_{2}\left(G\right)|=16\times 2^{n}-18$ and $|E_{3}\left(G\right)|=6\times 2^{n}-6$. From (2), the *F*-polynomial of *G* is calculated as

$$\begin{array}{cc} F(G,x) & =\sum_{uv\in E_{1}\left(G\right)}x^{\left[{\left(d_{u}\right)}^{2}+{\left(d_{v}\right)}^{2}\right]}+\sum_{uv\in E_{2}\left(G\right)}x^{\left[{\left(d_{u}\right)}^{2}+{\left(d_{v}\right)}^{2}\right]}+\sum_{uv\in E_{3}\left(G\right)}x^{\left[{\left(d_{u}\right)}^{2}+{\left(d_{v}\right)}^{2}\right]}\\ & =\sum_{uv\in E_{1}\left(G\right)}x^{5}+\sum_{uv\in E_{2}\left(G\right)}x^{8}+\sum_{uv\in E_{3}\left(G\right)}x^{13}\\ & =2^{n+1}x^{5}+\left(16\times 2^{n}-18\right)x^{8}+\left(6\times 2^{n}-6\right)x^{13}. \end{array}$$

 ☐

## 3. F-Index and Polynomial of Porphyrin Dendrimers

**Figure 2:**
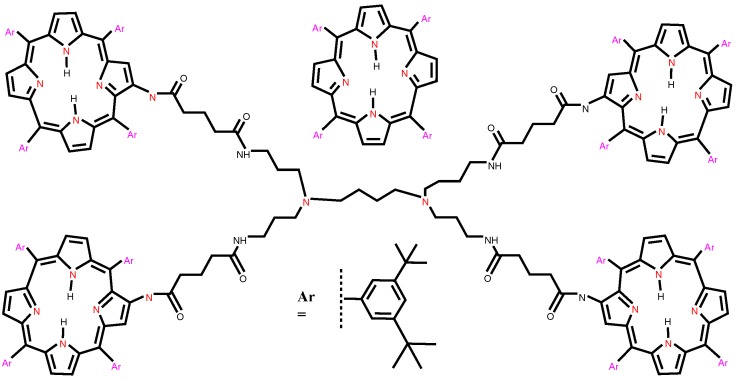
Molecular structure of porphyrin dendrimer $D_{4}P_{4}$.

**Figure 3:**
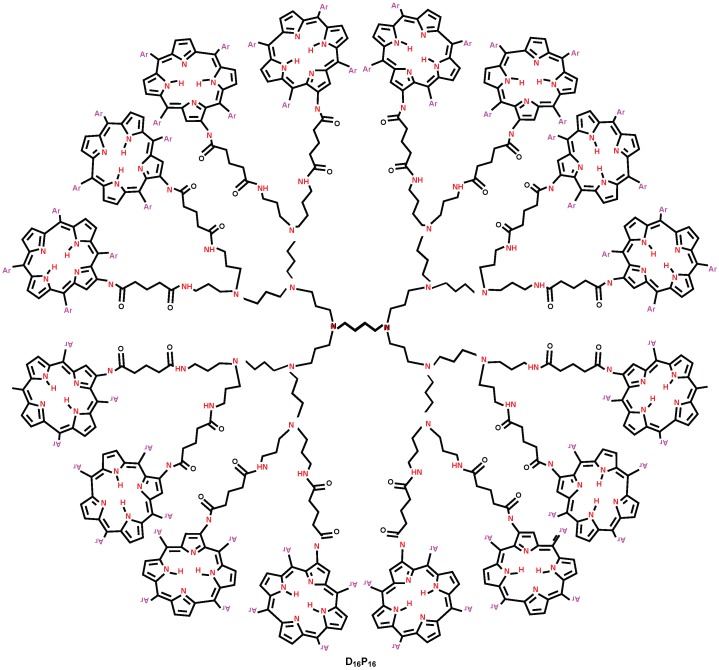
Molecular structure of porphyrin dendrimer $D_{16}P_{16}$.

We consider the class of porphyrin dendrimers, denoted by $D_{n}P_{n}$. Note that $n=2^{m}$, where m≥2 is steps of growth (see Figure 2). The molecular graph of $D_{n}P_{n}$ has four similar branches and a central core consisting of five extra edges (Figure 2 and Figure 3). In each branch of $D_{n}P_{n}$, we have $4+2\times 4+2^{2}\times 4+\cdots +2^{m-2}\times 4+2^{m-2}\times 88=24n-4$ vertices, among which $2^{m-2}\times 26$ vertices are of degree 1, $3+2\times 3+\cdots +2^{m-2}\times 3+2^{m-2}\times 28=17\times 2^{m-1}-3$ vertices are of degree 2, $8\times 2^{m-2}$ vertices are of degree 4, and the remaining $24n-4-2^{m-2}\times 26-\left(17\times 2^{m-1}-3\right)-8\times 2^{m-2}=7n-1$ vertices are of degree 3. Additionally, the central core contains four vertices of degree 2 and two vertices of degree 3. Therefore, in $D_{n}P_{n}$, there are a total of 96n-10 vertices, among which 26n vertices are of degree 1, 34n-8 vertices are of degree 2, 28n-2 vertices are of degree 3, and the remaining 8n vertices are of degree 4. It is easy to see from Lemma (1) that the total number of edges of $D_{n}P_{n}$ are 105n-11.

Since the molecular graph of $D_{n}P_{n}$ has four similar branches and five extra edges (Figure 2 and Figure 3), in which we have $e_{13}=4e_{13}^{\prime }$, $e_{14}=4e_{14}^{\prime }$, $e_{22}=4e_{22}^{\prime }+3$, $e_{23}=4e_{23}^{\prime }+2$, $e_{33}=4e_{33}^{\prime }$, and $e_{34}=4e_{34}^{\prime }$. By a routine calculation, we have $e_{13}^{\prime }=\frac{n}{2}$, $e_{14}^{\prime }=6n$, $e_{22}^{\prime }=5\times \frac{n}{2}-2$, $e_{23}^{\prime }=12n-2$, $e_{33}^{\prime }=13\times \frac{n}{4}$, and $e_{34}^{\prime }=4n$. It is easy to compute $e_{13}=2n$, $e_{14}=24n$, $e_{22}=10n-5$, $e_{23}=48n-6$, $e_{33}=13n$, and $e_{34}=8n$.

Now, we compute the F-index and polynomial of this type of dendrimer through the following theorem.

**Theorem** **2.** *Let $D_{n}P_{n}$ be a Porphyrin dendrimer. Then*

$$F(D_{n}P_{n})=1566n-118$$

$$F\left(D_{n}P_{n},x\right)=2nx^{10}+24nx^{17}+\left(10n-5\right)x^{8}+\left(48n-6\right)x^{13}+13nx^{18}+8nx^{25}$$

**Proof.**  Here we can use (1) and (2) and compute the *F*-inex and polynomial of porphyrin dendrimers in similar fashion as in Theorem 1. ☐

## 4. F-Index and Polynomial of Zinc–Porphyrin Dendrimer

**Figure 4:**
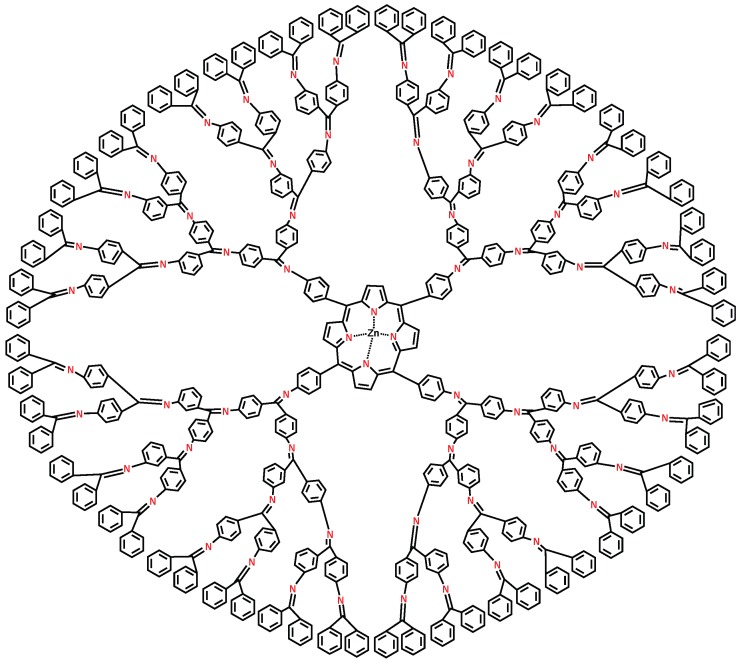
Molecular structure of dendrimer zinc porphyrin $DPZ_{4}$.

We consider the class of *dendrimer zinc–porphyrin $DPZ_{n}$* (see Figure 4), where *n* is the steps of growth and n≥1. The molecular graph of $DPZ_{n}$ consists of four similar branches and a central core. It is easy to see that the central core of $DPZ_{n}$ consists of 49 vertices among which 24 vertices are of degree two and three, respectively, and one vertex of degree four. In each branch of $DPZ_{n}$, we have $14+2\times 14+\cdots +2^{n-1}\times 14=14\left(2^{n}-1\right)$ vertices, among which $9+2\times 9+\cdots +2^{n-2}\times 9+2^{n-1}\times 11=11\times 2^{n}-9$ vertices are of degree two and the remaining $14\left(2^{n}-1\right)-\left(11\times 2^{n}-9\right)=3\times 2^{n}-5$ vertices are of degree three. Therefore, in $DPZ_{n}$, there are a total of $56\times 2^{n}-7$ vertices, among which $44\times 2^{n}-12$ vertices are of degree 2, $12\times 2^{n}+4$ vertices are of degree 3, and the remaining 1 vertex is of degree 4. It is easy to see from Lemma (1) that the total number of edges of $DPZ_{n}$ are $64\times 2^{n}-4$. Additionally, we can calculate $e_{22}=16\times 2^{n}-4$, $e_{23}=40\times 2^{n}-16$, $e_{33}=8\times 2^{n}+12$, and $e_{34}=4$. Now, we compute the *F*-index and polynomial of zinc–porphyrin dendrimer as shown in Figure 4.

**Theorem** **3.** *Let $DPZ_{n}$ be a zinc–porphyrin dendrimer. Then*

$$F\left(DPZ_{n}\right)=792\times 2^{n}-428$$

$$F\left(DPZ_{n},x\right)=\left(16\times 2^{n}-4\right)x^{8}+\left(40\times 2^{n}-16\right)x^{13}+\left(8\times 2^{n}+12\right)x^{18}+4x^{25}$$

**Proof.**  The proof is analogous to Theorems 1 and 2. ☐

## 5. Conclusions

In this paper, we dealt with three dendrimer families and studied *F*-index and *F*-polynomial on these molecular structures which will be helpful in computational chemistry. Moreover, we have also computed the edge partition of each dendrimer structure based on end vertices of each edge, which can be used to compute many other topological indices, as computed by the author in [18].

## Abbreviations

The following abbreviations are used in this manuscript:

PETIM	Poly(Propyl) Ether Imine
$D_{n}P_{n}$	Porphyrin dendrimers
DPZ	dendrimer Zinc-Porphyrin
